# Herniation of a Meckel’s diverticulum in the Umbilical Cord

**Published:** 2014-10-20

**Authors:** Ben Gys, Daphnée Demaeght, Guy Hubens, Martin Ruppert, Wouter Vaneerdeweg

**Affiliations:** 1 Department of Surgery, AZ St Dimpna, Geel, Belgium; 2 Department of Pediatrics, University Hospital Antwerp, Belgium; 3 Department of Abdominal Surgery, University Hospital Antwerp, Belgium

A female neonate born at term with a mass at the umbilicus (Fig. 1). Prenatal ultrasound at 22 weeks gestation had noted polyhydramnios, macrosomia, a single umbilical artery and a small ventricular septum defect. Fluorescence in situ hybridization (FISH) analysis at 27 weeks gestation did not identify any chromosomal abnormalities. At birth, the umbilical ring seemed intact and there was no real abdominal wall defect. Initially, a manual reduction of the hernia was established, yet it reoccurred almost immediately. Surgical exploration revealed a herniated Meckel’s diverticulum in the umbilical cord. 

Umbilical cord herniation is a rare (approximately 1-5000 live births) embryopathy, which is sporadically associated with other congenital anomalies [1]. The herniation is caused by the failure of the return of midgut into the celomic cavity at 10-14 weeks amenorrhea [2]. The coverings of the hernia are a thin membrane (Rathke’s membrane – continuous with the parietal peritoneum), Wharton’s jelly and a thin amniotic layer. There is no major abdominal wall defect with normal insertion of the rectus muscles in the xiphoid and the umbilical ring is intact. These are not the case in an omphalocele. As the ring might be too narrow to allow a spontaneous reduction, herniation of intra-abdominal organs could result in ischemia and necrosis. For that reason careful examination is vital. Furthermore, postnatal clamping of a herniated cord could result in an iatrogenic injury. Nowadays the umbilical cord herniation is mostly diagnosed during early prenatal screening [3]. This helps in pre- and postnatal decision making. The major criterion for differentiating a congenital hernia from an omphalocele is the morphology of the umbilical cord insertion i.e. the intactness of the umbilical ring. Herniation of the umbilical cord is sporadically associated with anomalies like an ileal atresia, cloacal anomalies or like in this case a persistent vitello-intestinal duct [4, 5].

**Figure:**
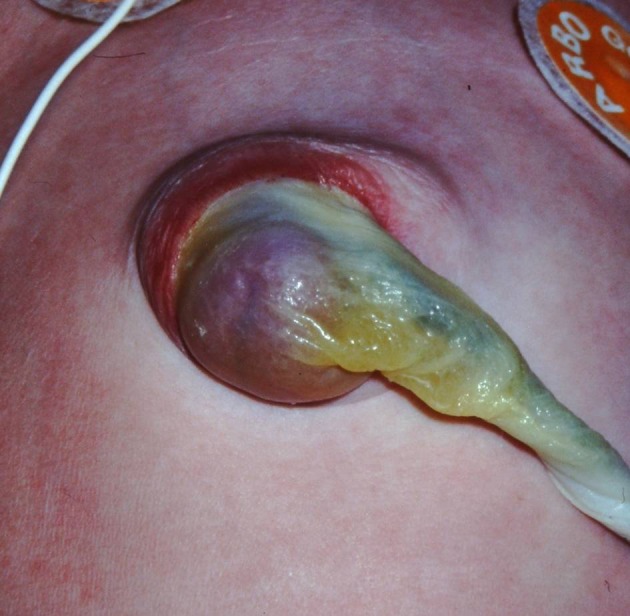
Figure 1: Impression of the umbilical cord herniation.

## Footnotes

**Source of Support:** Nil

**Conflict of Interest:** Nil

